# Perceived mistreatment in patients with rheumatic diseases: The impact of the underlying diagnosis

**DOI:** 10.1371/journal.pone.0316312

**Published:** 2024-12-30

**Authors:** Irazú Contreras-Yáñez, Loraine Ledón-LLanes, Guillermo Arturo Guaracha-Basañez, América Sánchez-Hernández, Ana Belén Ortiz-Haro, Virginia Pascual-Ramos

**Affiliations:** 1 Department of Immunology and Rheumatology, Instituto Nacional de Ciencias Médicas y Nutrición Salvador Zubirán, Mexico City, Mexico; 2 Department of Biology of Reproduction, Instituto Nacional de Ciencias Médicas y Nutrición Salvador Zubirán, Mexico City, Mexico; Murdoch University, AUSTRALIA

## Abstract

**Background:**

Mistreatment is a complex problem that impacts people’s quality of life, morbidity, and mortality. In aged people, it has been associated with female sex, poor general health, depression, functional and cognitive decline, and increased dependence levels, all of which are well-recognized characteristics of patients with rheumatic diseases (RMDs). The objective was to describe the mistreatment phenomenon in Mexican patients with RMDs. We additionally report the adaptation and validation of the Geriatric Mistreatment Scale (GMS) in the target population.

**Patients and methods:**

This cross-sectional study was developed in two phases (June 28, 2023-February 2, 2024), and three convenience samples were used: S-1 (n = 30), S-2 (n = 260), and S-3 (n = 372). *Phase 1* consisted of adapting the GMS to RMDs (RMD-MS) (experts’ agreement), followed by RMD-MS face validity (pilot testing, S-1), content validity (experts’ agreement), concurrent criterion validity (family APGAR score ≤3, S-2), construct validity (exploratory factor analysis and convergent validity, S-2), reliability (internal consistency and temporal stability, S-2) and feasibility (in S-1). *Phase 2* consisted of the mistreatment description in S-3.

**Results:**

Patients represented typical RMD outpatients with substantial disease duration. There were 187 (50.3%) patients with overall mistreatment, and psychological was the most frequent in 142 (75.9%) patients, followed by neglect mistreatment in 96 (51.3%), sexual in 30 (16%), physical in 23 (12.3%), and economic mistreatment in 20 (10.7%) patients. Patients’ perceived mistreatment was related to the underlying RMD in 13.3% of sexual mistreatment and 53.3% of psychological mistreatment. The number of "I do not want to answer" responses raised to 21.7%-67.7% for abusers’ sex and 40%-72.9% for the abusers’ relationship with the participant. The RMD-MS was valid, reliable, and feasible.

**Conclusions:**

Half of the Mexican patients with RMDs perceived some mistreatment, most frequently psychological mistreatment, which is also often perceived as related to the underlying RMD.

## Introduction

Rheumatic diseases (RMDs) integrate a complex group of chronic musculoskeletal conditions primarily characterized by musculoskeletal pain, impaired function and quality of life (QoL), comorbidity coexistence, and overall increased mortality [1–3]. RMDs interfere with patient’s ability to perform social roles and work productivity and translate into work disability over the years in a substantial proportion of affected patients [4, 5]. In addition, the full spectrum of RMDs’ clinical expression evidences that patients might present with progressive health decline, disability, and distressing symptoms, halting their capacity to continue with usual activities and roles, limiting patient autonomy, and favoring patients’ dependence on careers [6].

The etiology of RMDs is multifactorial, with genetic, environmental, hormonal, and immunological factors considered relevant in their development [7]. However, unknown trigger factors are recognized in half of the cases. Epidemiological research increasingly suggests that exposure to traumatic stressors and psychological trauma is related to increased healthcare utilization, impaired health, the onset of specific diseases, and premature death [7–9]. The association has been confirmed among patients with RMDs. Adverse experiences and stressors, especially during childhood and adolescence, have been suggested to play a role as possible causes, linked to negative outcomes, and have been proposed to influence the course of RMDs [10–25].

Mistreatment and related forms of abuse and violence are complex problems that impact the landscape of public health, criminal justice, and human rights [26]. In medical literature, mistreatment, abuse, and violence are often used interchangeably. The World Health Organization (WHO) defines violence as "The intentional use of physical force or power, threatened or actual, against oneself, another person, or against a group or community, that either results in or has a high likelihood of resulting in injury, death, psychological harm, maldevelopment, or deprivation" [26]. The WHO categorizes violence into three types based on the perpetrator: self-directed violence, interpersonal violence (further divided into family and intimate partner violence), and collective violence. Additionally, the WHO identifies four natures of violence within this analytical framework: physical, psychological, sexual, and deprivation/neglect, with each nature affecting each type of violence, except for self-directed violence, which does not recognize the sexual nature [26].

Violence is frequently regarded as an unavoidable aspect of the human condition, something to be reacted to rather than prevented [26]. However, driven by the success of public health approaches to other environmental and behavioral health issues, these assumptions are currently evolving [26]. The incidence of interpersonal violence, particularly among the elderly, is increasingly recognized in the healthcare system as a result of population aging [27]. This form of violence, which will be referred to as mistreatment, is recognized for its impact on people’s quality of life, morbidity, and mortality. In the elderly, it has been associated with female sex, poor self-reported general health, depression, functional and cognitive decline, and increased dependence levels [27–30], all of which are well-recognized characteristics of patients with RMDs. Despite this, there is limited published evidence of any form of violence, as comorbid conditions of RMDs [12, 17, 19, 20, 22] and their relationship with the underlying diagnosis have not been explored. An accurate estimate of this phenomenon is essential for its visibility and intervention planning and has ethical implications.

With the above considerations in mind, the primary objective of the current study was to estimate mistreatment and to describe its patterns in Mexican patients with RMDs. We additionally described the adaptation of the Geriatric Mistreatment Scale (GMS) to RMDs and its validation in the target population.

## Materials and methods

### Ethics

The study was performed in compliance with the Helsinki Declaration [31]. The Research Ethics Committee of the Instituto Nacional de Ciencias Médicas y Nutrición Salvador-Zubirán (INCMyN-SZ) approved the study (Reference number: IRE-4538-23-24-1), which was registered on clinicaltrial.gov (NCT06233760). All the patients provided written informed consent.

### Study design and period, setting, and study population

This cross-sectional study was performed in two phases between June 28, 2023, and February 2, 2024. The first phase was planned to develop an instrument with convenient psychometric properties to assess the primary objective. This objective, to estimate the prevalence of mistreatment in Mexican patients with RMDs, was achieved during phase 2.

Phase 1 (June 28, 2023-November 30, 2023) consisted of adapting the GMS to patients with RMDs, followed by the validity and reliability of the adapted version (the RMD-MS). Briefly, the GMS was created to assess the mistreatment of older people in a Spanish-speaking population from Mexico, where it has shown satisfactory results in terms of its psychometric properties [28]. The GMS was initially administered to 626 individuals aged 60 years or older, using a probabilistic sample that was representative of older adults living in Mexico City. Among them, 36.4% reported depression, 32% reported subjective memory complaints, and 56.1% reported poor general health. Additionally, 10.3% of the target population reported dependence on one or more activities of daily living [28]. The original version included 22 items distributed across five dimensions (physical, psychological, neglect, economic, and sexual), and each of the items allowed a dichotomous-type response (no occurrence/occurrence). Authors from the original scale considered each item to include an act of mistreatment in the past year and proposed a score with the 22-item scale (general mistreatment) or specifically for the type of mistreatment (according to the five dimensions). Higher scores of the GMS translated into more acts of mistreatment (S1 Appendix). Phase 1 also included the pilot testing for RMD-MS face and content validity and feasibility, followed by its construct and criterion validity and reliability. Guidelines for the development and validation of patient-reported outcome measures were followed [32].

During Phase 2 (from December 1, 2023, to February 2, 2024) we focused on assessing mistreatment in the target population, examining patterns and how patients perceive mistreatment in relation to their underlying RMDs. To achieve this, we utilized the previously validated version of the RMD-MS to survey outpatients with RMDs.

### Description of samples and sample size calculation

Inclusion criteria were patients with a diagnosis of an RMD based on the primary rheumatologist’s assessment, who have a scheduled consultation at the outpatient clinic, and who have provided written informed consent. Exclusion criteria included patients receiving palliative care, those identified by their primary rheumatologist as having a short-term prognosis with high mortality risk, and individuals with overlap syndrome—except for those with Sjögren syndrome. Patients were included in the study using convenience sampling, a non-probability method that selected participants available at the outpatient clinic [33].

**Fig 1:**
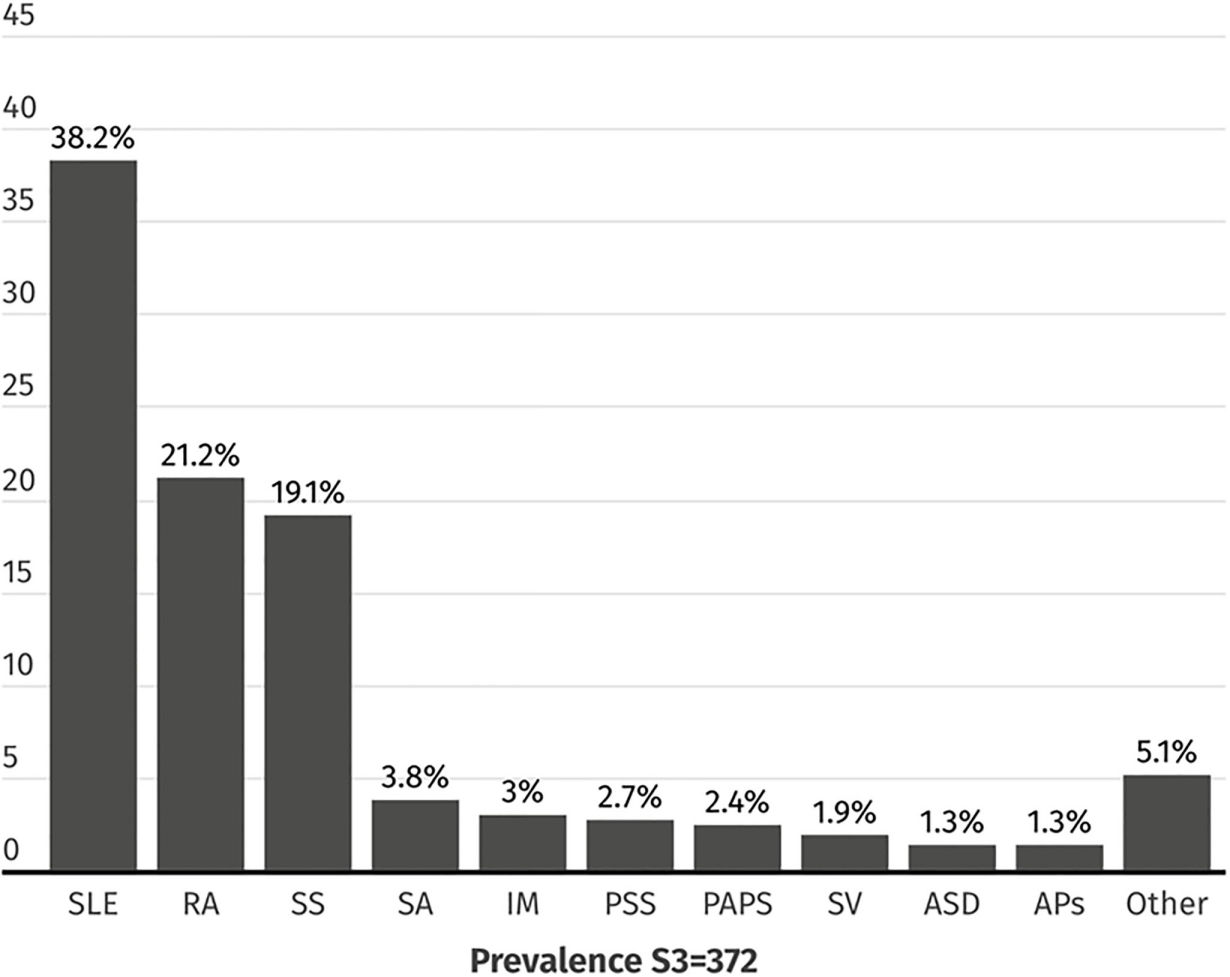
RMD diagnoses distribution in the sample used for estimating mistreatment. Data are presented as number of patients, %. SLE = Systemic Lupus Erythematosus. RA = Rheumatoid Arthritis. SS = Systemic Sclerosis. SA = Spondyloarthritis. IM = Inflammatory Myopathies. PSS = Primary Sjögren Syndrome. PAPS = Primary Anti-phospholipid Syndrome. SV = Systemic Vasculitis. ASD = Adult Onset-Still disease. APs = Psoriatic Arthritis.

**Fig 2:**
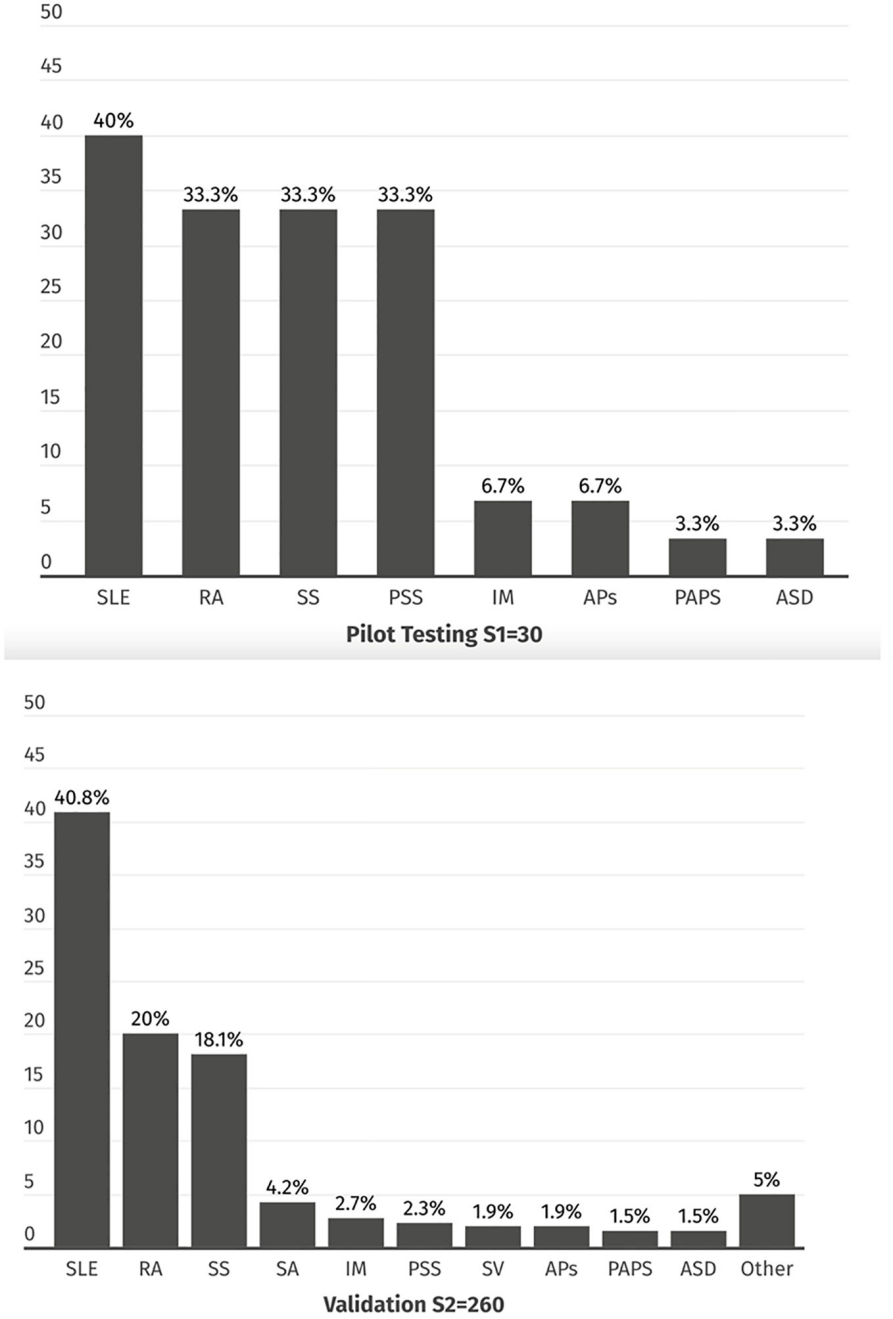
RMD diagnoses distribution in the samples used during the GMS adaptation to RMDs and the GMS validation. Data are presented as number of patients, %. SLE = Systemic Lupus Erythematosus. RA = Rheumatoid Arthritis. SS = Systemic Sclerosis. SA = Spondyloarthritis. IM = Inflammatory Myopathies. PSS = Primary Sjögren Syndrome. PAPS = Primary Anti-phospholipid Syndrome. SV = Systemic Vasculitis. ASD = Adult Onset-Still disease. APs = Psoriatic Arthritis.

Three samples of outpatients were used, and quotes (number of patients with a particular RMD diagnosis) were considered to represent the distribution of the ten most frequent local diagnoses (Figs 1 and 2).

Two samples were considered during Phase 1 as recommended [34–36]: the first one (S-1) included 30 outpatients who participated in pilot testing, and the second sample (S-2) included 260 outpatients. Both samples were used for RMD-MS validation. The third sample (S-3) included 372 patients with RMDs and was used during phase 2 to achieve the primary objective.

We estimated the sample size to detect (general) mistreatment, considering a prevalence of 48% of mistreatment according to data from Castro et al. [12], who investigated abuse in 58 patients with fibromyalgia, 74 with rheumatoid arthritis (RA), and 55 with soft tissue rheumatic disease. Before the pandemic, around 7.600 patients were coded with a definite RMD within the Department of Immunology and Rheumatology’s database. The final required estimated sample size was (at least) 365 patients, with a 95% confidence level and 5% precision.

### Procedures

Participants were recruited at the Department of Immunology and Rheumatology outpatient clinic of the INCMyN-SZ, an academic and national referral center for RMDs in Mexico City. Outpatients who arrived at the outpatient clinic were approached by a co-investigator who briefly explained the study’s aim and explored patients’ interest in participating. The informed consent process was performed once patients confirmed their interest (90% of the patients invited agreed to participate in the study). The following questionnaires and scales were administered in a standardized order: the RMD-MS was first applied, then the Routine Assessment of Patient Index Data (RAPID-3) [37], the Health Assessment Questionnaire Disability Index (HAQ-DI) [38], the World Health Organization QoL-Brief questionnaire (WHOQOL-BREF) [39], the family Adaptation, Partnership, Growth, Affection, and Resolve index (APGAR) [40] and the Depression, Anxiety, and Stress Scale (DASS21) [41], last applied. A brief description of the questionnaires and scales is presented in S2 Appendix.

#### Phase 1 procedures: GMS’s adaptation to RMDs, RMD-MS face, content, criterion and construct validity, and RMD-MS reliability [42]

Judgment experts adapted the GMS to RMDs. Two were rheumatologists, and one each was a psychologist and an MD, all involved in caring for patients with RMDs. In particular, for each dimension, an item was added to direct patients associating mistreatment (when applicable) to the underlying RMD or its impact on their lives and families (Yes/No/I don’t know). Modifications were incorporated after a consensus was reached (S1 Appendix).

RMD-MS face validity was confirmed during pilot testing with 30 patients with RMDs [42]. Judgment experts determined the RMD-MS content validity. The expert committee consisted of 10 members, including four rheumatologists, two bioethicists, three mental health specialists, and one social worker. They were selected based on their experience, reputation, availability, and impartiality, as recommended [43]. Experts independently rated 25 items according to the presence or absence of relevance, appropriate wording, and meaning. Experts also rated the instructions’ clarity [42].

For RMD-MS criterion validity, we considered a severely dysfunctional family (defined with a family APGAR score ≤3) as a criterion to assess concurrent validity [42].

The construct validity of the RMD-MS was established through convergent validity, which involved examining the correlations between the RMD-MS and several other assessments. These included the RAPID-3 score, a surrogate measure of perceived overall health related to disease activity and severity of the underlying RMD; the HAQ-DI, which reflects functional decline and dependency; and the WHOQOL-BREF, which is significantly influenced by overall health and experiences of mistreatment. Additionally, the correlations with DASS21 scores, which assess mental health, including (but not limited to) levels of depression, further supported the construct validity of the RMD-MS. This validity was also confirmed through factor analysis [42].

RMD-MS reliability was assessed with internal consistency and temporal stability (test-retest), which was tested after the questionnaire was applied to 50 patients, twice, at baseline and one week apart.

Finally, the RMD-MS feasibility was examined in the 30 patients participating in the pilot testing according to the following criteria: clarity of instructions and items, convenience of the questionnaire, and patient acceptance of the format.

#### Phase-2 procedures

The RMS-MS was administered, and standardized formats were used to assess relevant sociodemographic and disease-related variables, comorbid conditions based on the Rheumatic Diseases Comorbidity Index [44], and treatment-related variables, summarized in Table 1.

**Table 1:** Description of the patients in the sample used for assessing the primary objective (Mistreatment and patterns).

	S-3 n = 372
**Sociodemographic characteristics**	
Female sex*	345 (92.7)
Years of age	45 (33–54)
Years of formal education	12 (9–16)
Formal and non-formal job*	153 (41.1)
Married or Living with a partner*	201 (54.1)
Religious beliefs*	309 (83.1)
Medium-low socioeconomic status*	338 (90.9)
Family APGAR score (0–10 scale)	10 (8–10)
Patients with normal family function*	307 (82.5)
**Disease related-variables**	
Years of disease duration	10 (5–17)
RAPID-3 score (0–30 scale)	6.5 (1.3–12)
Patients with remission (RAPID-3 ≤3)	136 (36.6)
HAD-DI score (0–3 scale)	0.25 (0–1)
Patients with disability (HAQ-DI score >0.5)*	140 (37.6)
WHOQOL-BREF physical health score	50 (39.3–60.7)
WHOQOL-BREF mental health score	58.3 (50–70.8)
WHOQOL-BREF social relationship score	50 (50–75)
WHOQOL-BREF environment score	53.1 (46.9–62.5)
Rheumatic Diseases Comorbidity Index score	0 (0–1)
Patients with ≥ 1 comorbid condition*	185 (49.7)
One year-previous hospitalizations*	78 (21)
**Treatment-related variables**	
Immunosuppressive treatment*	315 (84.7)
N° of immunosuppressive drugs /patient^1^	2 (1–2)
Corticosteroids use*	143 (38.4)
**Mental health comorbidity**	
DASS21 score of ≥moderate severity*^,^^2^	
*Depression*	60 (16.1)
*Anxiety*	82 (22)
*Stress*	61 (16.4)

Data presented as median (Q25-Q75) as otherwise indicated.

*Number (%) of patients.

^1^Among those who met the characteristic.

^2^Lovibond SH, Lovibond PF. Manual for the Depression Anxiety & Stress Scales. 2nd ed. Sydney: Psychology Foundation; 1995.

### Statistical analysis

The RMD-MS score was calculated as the sum of the individual item’s score (min 0-Max 25), and the final score is presented on a scale of 0 to 10. For mistreatment prevalence, we proposed that the five dimensions be scored with at least 1 item with a dichotomous response (Yes/No) to ensure the mistreatment construct is assessed (14 patients did not meet this criterion and were not included in S-3).

Descriptive statistics were performed to describe the variables of the patients included in the three samples, with frequencies and percentages for categorical variables or the mean/median and standard deviation (SD)/Q25-Q75 for continuous variables with normal/non-normal distribution.

Appropriate tests were used to compare variables between groups. The X^2^ test for categorical variables and the Mann-Whitney U test for continuous variables.

Face validity was examined with the Cohen Kappa Index and was considered acceptable ≥ 0.60 [42].

Content validity was examined with agreement percentages. Lawshe/Tristan’s content validity ratio was calculated for individual items and the RMD-MS (mean of individuals’ content validity ratios) [42, 45].

Concurrent validity (criterion validity) was examined by comparing RMD-MS general and domain scores and mistreatment prevalence between RMD patients with and without family APGAR score ≤3, with appropriate tests.

Convergent validity was analyzed using the Spearman rank correlation coefficient (rho) [34]. Construct validity was evaluated using exploratory factorial analysis (principal components) with Varimax rotation. Sampling adequacy was confirmed using the Kaiser‐Mayer‐Olkin (KMO) (appropriate value ≥0.5) measure, and the use of factor analysis was supported by Bartlett’s test of sphericity (significant value p<0.05). The number of factors was defined as in the original GMS (five factors) [46].

To ensure the reliability of the questionnaire, we employed Cronbach’s α to assess its internal consistency. Furthermore, we conducted additional analyses by removing individual items, confirming that the RMD-MS successfully preserved the essential clinometric properties of the original instrument. For temporal stability/test-retest, intra-class correlation coefficients (ICC) and their 95% confidence intervals (CI) were calculated using a single measurement, absolute-agreement, 2-way mixed-effects model. Cronbach’s α, ICC, and 95% CI interpretations followed published recommendations [47]. Finally, the floor and ceiling effects of the questionnaire were determined as the percentage of patients who achieved the lowest and highest scores on the scale, respectively.

There were no missing data.

All statistical analyses were performed using Statistical Package for the Social Sciences version 21.0 (SPSS Chicago IL). A value of p<0.05 was considered statistically significant.

## Results

### Study population characteristics

The population for which we estimated the prevalence of mistreatment included 372 patients with RMDs. The distribution of their diagnoses and patients’ characteristics is summarized in Fig 1 and Table 1. Diagnosis quotes were represented, and the most frequent diagnoses were systemic lupus erythematosus (SLE) and RA (Fig 1). Patients were primarily middle-aged (45 [33–54]) women (345 [92.7%]) living with a partner (201 [54.1%], referred to religious beliefs (mainly Catholics) (309 [83.1%]), and had a normal family function based on the family APGAR score (307 [82.5%]). Overall, patients had substantial disease duration (10 years [5–17]), mild disease activity of the underlying RMD (RAPID-3 score: 6.5 [1.3–12]), and HAQ-DI score that translated into the absence of disability (140 [37.6%]), although QoL dimensions were compromised. Regarding treatment, most patients received immunosuppressive drugs (315 [84.7%]), and a significant proportion received corticosteroids (143 [38.4%]). Finally, half of the patients had some comorbidity, and a substantial number had current psychological comorbidity of at least moderate severity [39]: 16.1% had depression, and 22% had anxiety.

### Prevalence of (general) mistreatment and patterns in patients with RMDs

In S-3, the median (Q25-Q75) RMD-MS score was 1 (0–2). There were 187 (50.3%) patients with RMD-MS score ≥1, which corresponded to the prevalence of (overall) mistreatment. Among them, psychological was the most frequent mistreatment in 142 (75.9%) patients, followed by neglect mistreatment in 96 (51.3%), sexual in 30 (16%), physical in 23 (12.3%), and economic mistreatment in 20 (10.7%). Overall, the majority of the patients had one single mistreatment pattern (N = 111 [59.4%]), 41 had two (21.9%), 24 had three (12.8%), 9 had four (4.8%), and two patients (1.1%) scored the five mistreatment patterns. Table 2 summarizes the response distribution for each item. The percentage of "I do not want to answer" responses was below 1%.

**Table 2:** Mistreatment patterns: Item responses distribution (n° [%]).

	Yes	No	I don´t know	I don´t want to answer	It does not apply to me
**Physical**					
Have you been hit, for instance, by punches or kicks?	10 (2.7)	360 (96.8)	0	0	2 (0.5)
Have you been shoved, shacked, or had your hair pulled?	19 (5.1)	350 (94.1)	0	0	3 (0.8)
Have you had an object thrown at you intended to hurt you?	3 (0.8)	367 (98.7)	0	0	2 (0.5)
Have you been assaulted with a knife, blade, gunfire, or another object?	4 (1.1)	366 (98.4)	0	0	2 (0.5)
**Psychological**					
Have you felt humiliated or made fun of?	90 (24.2)	276 (74.2)	6 (1.6)	0	0
Have you felt ignored or treated with indifference?	86 (23.1)	281 (75.5)	5 (1.3)	0	0
Have you felt you have been isolated?	64 (17.2)	302 (81.2)	5 (1.3)	1 (0.3)	0
Has anyone made you feel afraid?	61 (16.4)	310 (83.3)	1 (0.3)	0	0
Has anyone made you feel less valued as a person?	68 (18.3)	300 (80.6)	4 (1.1)	0	0
**Neglect**					
In general, have your decisions been respected?	331 (89)	37 (9.9)	3 (0.8)	1 (0.3)	0
Even having the necessary conditions, has anyone refused to provide you with essential things (clothes, food…) when needed?	35 (9.4)	337 (90.6)	0	0	0
Even having the necessary conditions, has anyone refused to provide you with medications or required therapies when needed?	20 (5.4)	352 (94.6)	0	0	0
Even having the necessary conditions, has anyone denied you help to go to a medical consultation or therapy when needed?	23 (6.2)	347 (93.3)	0	1 (0.3)	1 (0.3)
Have you been denied protection even having the necessary conditions when you have felt that someone or something could harm you?	17 (4.6)	351 (94.4)	0	1 (0.3)	2 (0.5)
Have you been forbidden to go out or to be visited?	17 (4.6)	351 (94.4)	0	1 (0.3)	3 (0.8)
Have you been denied access to your home?	4 (1.1)	365 (98.1)	1 (0.3)	0	2 (0.5)
Have you been kicked out of the house?	15 (4)	355 (95.4)	0	1 (0.3)	1 (0.3)
**Economic**					
Has anyone managed or is anyone managing your money without your consent or pressing you to assign it to some family expenses?	1 (0.3)	368 (98.9)	0	0	3 (0.8)
Has your money been taken from you?	8 (22)	361 (97)	0	0	3 (0.5)
Has anyone taken any of your belongings without your permission?	13 (3.5)	353 (94.9)	2 (0.5)	0	4 (1.1)
Have any of your properties been sold without your consent?	13 (3.5)	365 (98.1)	1 (0.3)	0	5 (1.3)
Have you been pressured so that you no longer own your house or any other property?	2 (0.5)	365 (98.1)	0	0	5 (1.3)
**Sexual**					
Have you been forced to have sex even if you did not want to?	8 (2.2)	357 (96)	1 (0.3)	3 (0.8)	3 (0.8)
Has anyone touched your body, including your genitals, without your consent?	11 (3)	354 (95.2)	1 (0.3)	3 (0.8)	3 (0.8)
Have you felt sexual rejection from your partner?	15 (4)	343 (92.2)	2 (0.5)	3 (0.8)	9 (2.4)

Data presented as N° (%) of responses to the item.

Among patients who rated mistreatment, they perceived it was related to the underlying RMD or RMD impact on their lives and families in a variable percentage, from 13.3% for sexual mistreatment to 53.3% for psychological mistreatment (Table 3).

**Table 3:** Mistreatment relationship with the underlying RMD.

	Yes	No	I don´t know
**Physical**	9 (39.1)	11 (47.8)	3 (8.7)
**Psychological**	76 (53.5)	43 (20.3)	23 (16.2)
**Neglect**	19 (19.8)	24 (25)	53 (55.2)
**Economic**	7 (35)	8 (40)	5 (25)
**Sexual**	4 (13.3)	17 (56.7)	9 (30)

Data presented as N° (%) of responses to the question, “If you have provided a positive answer to at least one of the items, do you consider it related to the underlying rheumatic disease or its impact in your life and your family? “

Finally, patients with a positive answer to at least one mistreatment pattern were selected to identify the sex and the relationship with the abuser. The number of "I do not want to answer" responses raised to 21.7%-67.7% for sex identification and 40% to 72.9% for the relationship with the patient. Overall, men (for neglect, economic, and sexual mistreatment patterns, Table 4) and family members (all mistreatment patterns but for psychological mistreatment, Table 5) were frequently identified as abusers.

**Table 4:** Abuser sex (identified by the patient) among patients who scored at least one mistreatment.

	Male	Female	Both	I do not want to answer
**Physical (N = 23)**	8 (34.8)	7 (30.4)	3 (13)	5 (21.7)
**Psychological (N = 142)**	28 (19.7)	21 (14.4)	56 (39.4)	37 (26.1)
**Neglect (N = 96)**	16 (16.7)	10 (10.4)	5 (5.2)	65 (67.7)
**Economic (N = 20)**	9 (45)	4 (20)	1 (5)	6 (30)
**Sexual (N = 30)**	17 (56.7)	2 (6.7)	1 (3.3)	10 (33.3)

Data presented as the number (%) of patients who selected the response.

**Table 5:** Abuser relationship with the patient (identified by the patient) among patients who scored at least one mistreatment.

	Family member	Stranger	Couple	I do not want to answer
**Physical (N = 23)**	8 (34.8)	2 (8.7)	1 (4.3)	12 (52.2)
**Psychological (N = 142)**	32 (22.5)	45 (31.7)	9 (6.3)	58 (40.8)
**Neglect (N = 96)**	17 (17.7)	4 (4.2)	5 (5.2)	70 (72.9)
**Economic (N = 20)**	10 (50)	0	2 (10)	8 (40)
**Sexual (N = 30)**	7 (23.3)	3 (10)	6 (20)	14 (46.7)

Data presented as the number (%) of patients who selected the response.

### RMD-MS validation process

The validation process included 290 patients divided into two samples (S1 and S2). The distribution of diagnoses (Fig 2) and the patients’ characteristics (S1 Table) were similar to what was observed in the overall population, where the prevalence was estimated.

#### Adaptation process

S1 Appendix details all the relevant changes in italics. Experts proposed three new items, joined two items into one, split one into two, and assigned three items to different dimensions.

Experts also extended the scale response, and the following options were added: “I do not know,” “I do not want to answer,” and “It does not apply to my situation.”

Also, for each mistreatment dimension, one item was added and directed to explore if a positive answer to a mistreatment pattern was perceived as related to the underlying RMD: “If you have provided a positive answer to at least one of the items, do you consider it is related to the underlying rheumatic disease or its impact on your life and your family? ”

Finally, items directed to investigate the sex and relationship of the mistreatment’s responsible (when appropriate) were investigated for the whole dimension instead of each item.

#### Patients RMD-MS face validity

During pilot testing (S-1), all the patients agreed on face validity, and the Cohen Kappa Index was 1.

#### Experts RMD-MS content validity

Experts agreed on the items’ clarity (82% to 100% agreement), scale response adequacy (100% agreement), and instructions clarity (100% agreement). The individual content validity ratio varied from 0.8 to 1. The (mean) content validity ratio for the RMD-MS was 0.94.

#### RMD-MS criterion validity

S2 Table summarizes the results. Briefly, patients with normal family function scored lower on the RMD-MS and presented a lower prevalence of mistreatment than their counterparts.

#### RMD-MS construct validity

S3 Table summarizes Spearman rank correlation coefficients (rho) between the RMD-MS score, specific dimensions scores of the DASS21 (depression, anxiety, and stress), the HAQ-DI score, and the four dimensions of the WHOQOL-BREF. Overall, correlations were significant, but their strengths were poor.

The 25 items were distributed into five domains: physical, psychological, neglect, economic, and sexual. The KMO measure was 0.795, and we observed a significant result (X^2^ = 5965.308, p≤0.001) for the Bartlett sphericity test. The 5-factor structure accounted for 68.6% of the variance (S4 Table).

#### RMD-MS internal consistency and reliability

Results of internal consistency (Cronbach’s α, ICC [95% CI]) of the RMD-MS and each domain are presented in Table 6, which additionally presents the floor and ceiling effects. S5 Table indicates that removing individual items did not negatively affect the global Cronbach’s α values for RMD-MS, nor the Cronbach’s α values for its dimensions.

**Table 6:** Psychometric characteristics dimensions that integrated the RMD-MS.

RMD-MS (N° of items; items location)	Cronbach´s α	ICC 95% CI	Mean of inter-item correlations	Floor/ceiling effects (%)
Physical (4; 1–4)	0.958	0.955 (0.946–0.962)	0.889	94.2 / 0.4
Psychological (5; 5–9)	0.857	0.857 (0.830–0.881)	0.642	62.7 / 5
Neglect (8; 10–17)	0.796	0.792 (0.755–0.826)	0.413	75 / 0.8
Economic (5; 18–22)	0.913	0.912 (0.895–0.927)	0.740	95.8 / 0
Sexual (3; 22–25)	0.817	0.785 (0.739–0.824)	0.732	91.9 / 0.4
RMD-MS (25)	0.888	0.885 (0.865–0.903)	0.683	49.6 / 0

Rho and ICC (95% CI) for temporal stability/test-retest were 0.903 and 0.930 (0.873–0.962), respectively. The mean (±SD) of the time between the two measurements in the test-retest analysis was 8 ± 1 days.

*RMD-MS feasibility*. During pilot testing (S-1), 26 (86.7%) patients agreed on the instruction’s clarity, and 29 (96.7%) each on the items’ clarity and format adequacy. Patients took (mean ± SD) 13.3±8.4 minutes to complete RMD-MS. Most patients (16 [53.3%]) considered the scale took time to fill out but were willing to do it.

## Discussion

The current study assessed mistreatment in patients with RMDs, using a previously adapted and validated questionnaire in the target population. Mistreatment is a complex phenomenon, and its measurement should ideally involve operationalizing a defined variable and developing and applying an instrument to its adequate quantification and to avoid misclassification bias.

First, half of the Mexican patients with RMDs scored on the RMD-MS for overall mistreatment. The most frequent mistreatment pattern was psychological mistreatment, followed by neglect, sexual, physical, and economic mistreatment. Patients attributed mistreatment to the underlying RMD (or RMD impact on their lives and families) in a variable percentage, from 13.3% for sexual mistreatment to 53.3% for psychological mistreatment. Mistreatment has been widely described, particularly in vulnerable elders, among whom nearly a quarter report significant levels of primarily psychological mistreatment [27]. In the realm of RMDs, a significant prevalence of mistreatment and any form of abuse has been documented, particularly among patients with fibromyalgia [12, 22, 24, 25, 48–51], RA [12], and more recently, among patients with SLE [52]. Two studies warrant special attention, as they were conducted in countries within the Latin American region. In a study by Castro et al. [12] 187 out of 500 new patients at a Guatemalan outpatient rheumatic clinic were examined, alongside 187 matched controls. The patient group included individuals with fibromyalgia (58), RA (74), and soft tissue rheumatic disease (55). Participants reported on experiences of verbal, physical, and sexual abuse via a questionnaire. Findings indicated significantly higher rates of physical and verbal abuse in patients compared to controls, particularly notable in fibromyalgia patients where abuse prevalence was 70.7%. RA and soft tissue rheumatic disease patients showed abuse prevalence of 35.1% and 41.8%, respectively, against only 15% in controls. Campos-Tinajero et al. [52] assessed the prevalence and impact of intimate partner violence (IPV) on the health-related QoL of 85 women with SLE. The study took place at an urban outpatient clinic in the North of Mexico between September 2022 and September 2023. The researchers found that one in four women had experienced IPV in the previous year, and those who were exposed to it had a diminished QoL. Additionally, they observed that the severity of the abuse was correlated with disease activity. Our prevalence of overall mistreatment is similar to that observed in the literature, where emphasis on verbal mistreatment (which is frequently included in the psychological pattern) is also reported. However, when patients were directed to detail mistreatment attribution to the underlying RMD (or its impact on their lives and families), mistreatment prevalence decreased differentially according to the mistreatment pattern and reached 53.5% for psychological mistreatment, while the attribution dropped to 13% for sexual mistreatment. These findings should be viewed in light of the high levels of violence in Mexico, as indicated by crime statistics related to life, physical well-being, freedom, sexual safety, and family well-being [53, 54]. We argue that mistreatment is a dynamic and contextual process at the individual level, presenting multiple layers, similar to the vulnerability layers proposed by Florencia Luna [55]. Mistreatment is not confined to a single form or layer; rather, multiple layers may coincide within the same individual. Therefore, a person may attribute their experience of abuse to family or partner dynamics, living conditions, an illness that makes them dependent, or a combination of these factors. Our findings do not support the idea of a one-way, exclusive link between mistreatment and chronic diseases. Although the questionnaire focused on the patient’s perspective, it may still be challenging for the patient to differentiate the factors contributing to the experience of mistreatment.

Second, we observed that patients who reported some mistreatment were reluctant to identify the sex and the relationship of the abuser, as evidenced by the increase in the "I do not want to answer" responses from less than 1% in the items identifying mistreatment and its patterns, to up to 72.9% when referring to the relationship with the abuser. This result might reflect the isolated and secretive nature of abusive acts and question if we are detecting the most severe phenomenon [27]. Previous studies have mentioned potential reasons why violence victims can silence their traumatic experiences, including the fear of retaliation and not being believed, but also perpetrators employing strategies to silence their victims [56]. Besides, there is often some dependency involved in abusive relationships, which leads to victims’ paradoxical responses aiming to sustain psychophysiological homeostasis and behavioral functioning [57].

We also observed that overall, men and family members were identified as abusers frequently, which has been replicated among Guatemalan patients with RMDs, who have cited family members as the most frequent abusers [12]. Meanwhile, the men’s sex/gender as the most frequently identified for neglect, economic, and sexual mistreatment brings into the analysis the patriarchal system funds and the traditional gender meanings (the population studied was integrated primarily by females). Gender-based violence is widespread and has different expressions, such as mistreatment. The ecological framework model explains this violence from the interplay of personal, situational, and sociocultural factors [58], and one dimension deeply involved is the cultural myth that settles masculine success as obtained through violence and dominance [59]. Gender-based violence has a life course development trajectory, relies on hegemonic masculinity-based identities, and links to all forms of violence against women and girls [60].

Third, the RMD-MS showed adequate psychometric properties. Face and content validity were examined by groups of experts that also included patients; clinicians may be the best observers for certain RMD aspects, but only patients can report on more subjective aspects [61]. The detected differences in RMD-MS general and dimension scores and the prevalence of mistreatment between patients with a normal family function and their counterparts favored criterion validity. The construct validity was demonstrated by KMO sampling and Barlett´s test of sphericity, both confirming the adequacy of the sample size for conducting factor analysis [46]; a five-factor structure was maintained, accounting for 68.6% of the variance. RMD-MS reliability was assessed with internal consistency and test-retest as recommended [34]; Cronbach´s α coefficient for the total scale and individual dimensions were good to excellent. These results were confirmed for ICC and 95% CI during test-retest, which was assessed in 50 patients. Finally, the RMD-MS was feasible based on patients’ evaluation during pilot testing and suitable for low-literacy patients.

Some limitations of the study need to be addressed. The study was conducted at a single academic center in a metropolitan area; patients included had different RMDs, although RA and SLE diagnoses were highly prevalent, which might impact the results’ generalizability. Also, the findings may be specific to the Latin American region due to the importance of the cultural context of the mistreatment construct. The RMD-MS showed a floor effect, which is defined as when more than 15% of the patients achieved the lowest score [35]; a floor effect can reduce the possibility of detecting change over time. Also, the study was cross-sectional, which limits the ability to infer causal relationships. RAPID-3 was used to assess disease activity/severity among patients with a wide variety of rheumatic diagnoses, while the scale has been validated only in RA patients. Finally, we determined a limited number of psychometric characteristics of the RMD-MS, which were considered necessary for a first approach; additional relevant factors, such as sensitivity to change, need to be defined.

## Conclusions

Half of the Mexican patients with RMDs reported experiencing mistreatment. Interestingly, patients didn’t always attribute the mistreatment to their underlying condition. The most common form of mistreatment reported was psychological, followed by neglect, sexual, physical, and economic mistreatment. Patients who reported mistreatment often did not disclose the gender or relationship of the abuser, but when they did, men and family members were frequently identified as the abusers. Additionally, our assessment tool, RMD-MS, demonstrated good psychometric properties for evaluating mistreatment among patients with RMDs.

These findings highlight the importance of identifying mistreatment in this patient population, which can help in planning appropriate actions. We are currently investigating the factors associated with mistreatment among patients with RMDs.

## Supporting information

S1 Appendix Original Geriatric Mistreatment Scale (GMS) and its adaptation to patients with rheumatic diseases (RMDs): Results from the judgment expert’s process. (PDF)

S2 Appendix Instruments´ description. (PDF)

S1 Table Characteristics of the patients who were included in the RMD-MS validation process (S-1 and S-2). (PDF)

S2 Table Comparison of RMS-MS general score and dimension scores and the prevalence of mistreatment between patients with a normal family function and their counterparts. (PDF)

S3 Table Spearman rank correlation coefficients (rho) between the RMD-MS score, specific dimensions scores of the DASS21, the family APGAR score, the HAQ-DI score, and the WHOQOL-BREF. (PDF)

S4 Table Factorial matrix. (PDF)

S5 Table Impact of removing items on RMD-MS Cronbach’s alpha. (PDF)
