# Supplementary material for: Perceived mistreatment in patients with rheumatic diseases: The impact of the underlying diagnosis
Source: PLoS One. 2024 Dec 30;19(12):e0316312. doi: 10.1371/journal.pone.0316312 (PMC11684605; doi:10.1371/journal.pone.0316312)
Supplement: S1 Appendix — (PDF) [file pone.0316312.s001.pdf]

Appendix 1. Original Geriatric Mistreatment Scale (GMS) and its adaptation to patients with rheumatic diseases (RMDs): Results from the judgment expert's process.

(C*hanges are highlighted in italics).*

GMS	RMDs-Mistreatment Scale
**Physical**	
1. Have you been?<br>2. Have you been punched or kicked?	1.- Have you been hit, for instance, by punches or kicks?<br>*Items 1 and 2 from GMS were combined*
3. Have you been shoved or had your hair pulled?	2.- Have you been shoved, shacked, or had your hair pulled?<br>*Corresponds to item 3 from the GMS. Shaked was added.*
4.- Have you had an object thrown at you?	3.- Have you had an object thrown at you intended to hurt you?<br>*Corresponding to item 4 from the GMS and intended to hurt you was added.*
5.- Have you been assaulted with a knife or blade?	4.- Have you been assaulted with a knife, blade, gunfire, or another object?<br>*Corresponds to item 5 from the GMS and …gunfire or any other object was added.*
**Psychological**	
6.- Have you been humiliated or made fun of?	5.- Have you felt humiliated or made fun of?<br>*Corresponds to item 6 from the GMS.*
7.- Have you been treated with indifference or ignored?	6.- Have you felt ignored or treated with indifference?<br>*Corresponds to item 7 from the GMS and "Have you been treated?" was updated "to have you felt?"*
8.- Have you been isolated or kicked out of the house?<br>*Item 8 from GMS was split into items, and one of them was moved to the Neglect dimension (item 17).*	7.- Have you felt you have been isolated?<br>*Corresponds to item 8 from the GMS and "Have you been treated?" was updated to "to have you felt?".*
9.- Has anyone made you feel afraid?	8.- Has anyone made you feel afraid?

	Corresponds to item 9 from the GMS.
10.- Have your decisions been respected?<br>*This item was moved to the Neglect dimension (item 10 from the RMD-MS).*	9.- Has anyone made you feel less valued as a person?<br>*Proposed as a new item*.
11.- Have you been forbidden to go out or be visited?<br>*This item was moved to the Neglect dimension (item 15 from the RMD-MS).*	
**Neglect**	
12.- Has anyone kept you from getting clothes, footwear, etc.?	10.- In general, have your decisions been respected?<br>*Corresponds to item 10 from the GMS.*<br>*Moved from the Psychological dimension of the GMS. In general, was added.*
13.- Has anyone kept you from receiving the medications you need?	11.- Even having the necessary conditions, has anyone refused to provide you with essential things (clothes, food…) when needed?<br>*Corresponds to item 12 from the GMS. "Even having the necessary conditions" and "When needed" were added.*
14.- Have you been denied protection when you need it?	12.- Even having the necessary conditions, has anyone refused to provide you with medications or required therapies when needed?<br>*Corresponds to item 13 from the GMS. "Even having the necessary conditions" and required therapies" were added.*
15.- Have you been denied access to the house where you live?	13.- Even having the necessary conditions, has anyone denied you help to go to medical consultation or therapy when needed?<br>*Proposed as a new item.*
	14.- Have you been denied protection even having the necessary conditions when you have felt that someone or something could harm you?<br>*Corresponds to item 14 from the GMS. Also, "having the necessary conditions" and "…someone or something could harm you" were added.*
	15.- Have you been forbidden to go out or to be visited?

	Corresponds to item 11 from the Psychological dimension.
	16.- Have you been denied access to your home?<br>*Corresponds to item 15 from the GMS. House where you live was updated to home.*
	17.- Have you been kicked out of the house?<br>*This partially corresponds to item 8 from the GMS, which was split into two items.*
**Economic**	
16.- Has anyone managed or is anyone managing your money without your consent?	18.- Has anyone managed or is anyone managing your money without your consent or pressing you to assign it to some family expenses?<br>*Corresponds to item 16 from the GMS and "pressing you to assign it to some family expenses" was added.*
17.- Has your money been taken from you?	19.- Has your money been taken from you?<br>*Corresponds to item 17 from the GMS.*
18.- Has anyone taken any of your belongings without your permission?	20.- Has anyone taken any of your belongings without your permission?<br>*Corresponds to item 18 from the GMS.*
19.- Have any of your properties been sold without your consent?	21.- Have any of your properties been sold without your consent?<br>*Corresponds to item 19 from the GMS.*
20.- Have you been pressured so that you no longer own your house or any other property?	22.- Have you been pressured so that you no longer own your house or any other property?<br>*Corresponds to item 20 from the GMS.*
**Sexual**	
21.- Have you been forced to have sex even if you did not want to?	23.- Have you been forced to have sex even if you did not want to?<br>*Corresponds to item 21 from the GMS.*
22.- Has anyone touched your genitals without your consent?	24.- Has anyone touched your body, including your genitals, without your consent?<br>*Corresponds to item 22 from the GMS. Your body was added.*
	25.- Have you felt sexual rejection from your partner?<br>*Proposed as a new item.*

Original Spanish version of the Geriatric Mistreatment Scale

Escala Geriátrica de Maltrato al adulto mayor

Se sabe que hay situaciones difíciles que normalmente no se platican pero que afectan mucho a las personas adultas mayores, conocer lo que pasa permitirá tomar las medidas necesarias para que en un futuro ya no suceda. Dígame si usted ha vivido alguno de los siguientes problemas en el último año, dentro o fuera del hogar.

	A<br>Si su respuesta es sí pase a B	B<br>¿Esto ocurrió...	C<br>¿Desde hace cuántos años ocurre esto?	D<br>Quién fue el responsable? PARENTESCO	E<br>¿Es hombre o mujer?
¿Durante los últimos 12 meses usted...	0 No<br>1 Sí*<br>* No respodio	1 una vez?<br>2 pocas veces?<br>3 mucha veces?<br>99 No respondió	1 Un año y menos<br>98 No recuerda	Registre el parentesco que tiene con la persona mayor	1 Hombre<br>2 Mujer
**FÍSICO**					
1. ¿Le han golpeado?					
2. ¿Le han dado puñetazos o patadas?					
3. ¿Le han empujado o le han jalado el pelo?					
4. ¿Le han aventado algún objeto??					
5. ¿Le han agredido con algún cuchillo o navaja?					
**PSICOLÓGICO**					
6. ¿Le han humillado o se han burlado de usted?					
7. ¿Le han tratado con indiferencia o le han ignorado?					
8. ¿Le han aislado o le han corrido de la casa?					
9. ¿Le han hecho sentir miedo?					
10. ¿No han respetado sus decisiones?					
11. ¿Le han prohibido salir o que la visiten?					
**NEGLIGENCIA**					
12. ¿Le han dejado de proporcionar la ropa, el calzado, etc?					
13. ¿Le han dejado de suministrar los medicamentos que necesita?					
14. ¿Le han negado protección cuando la necesita?					
15. ¿Le han negado acceso a la casa que habita?					
**ECONÓMICO**					
16. ¿Alguien ha manejado o maneja su dinero sin su consentimiento?					
17. ¿Le han quitado su dinero?					
18. ¿Le han tomado sin permiso algún bien de su propiedad?					
19. ¿Le han vendido alguna propiedad de su pertenencia sin su consentimiento?					
20. ¿Le han presionado para que deje de ser usted el propietario de su casa o de alguna otra propiedad?					
**SEXUAL**					
21. ¿Le han exigido tener relaciones sexuales aunque usted no quiera?					
22. ¿Le han tocado sus genitales sin su consentimiento?					

Total: ____ **/22**

Maltrato: Sí [1] No [0]

• Giraldo-Rodríguez, L., & Rosas-Carrasco, O. (2013). Development and psychometric properties of the Geriatric Mistreatment Scale. Geriatrics & Gerontology International, 13(2), 466–474. http://bit.ly/2zKM5qq.
