# Supplementary material for: Perceived mistreatment in patients with rheumatic diseases: The impact of the underlying diagnosis
Source: PLoS One. 2024 Dec 30;19(12):e0316312. doi: 10.1371/journal.pone.0316312 (PMC11684605; doi:10.1371/journal.pone.0316312)
Supplement: S2 Appendix — (PDF) [file pone.0316312.s002.pdf]

Appendix 2. Instruments´ description

RAPID-3

This index includes three measures: Physical function, pain, and a patient global estimate evaluation. It has a raw score of 0-30 and an adjusted score of 0-10, with higher scores translating into higher disease activity/severity. Four proposed categories are defined based on 0-30 scale cut-offs: >12 as high, 6.1-12.0 as moderate, 6.0-3.1 as low, and ≤3 remission [1].

HAQ-DI

This index includes 20 items that assess limitations to perform eight activities of daily living: dressing, arising, eating, walking, hygiene, reach, grip, and activities. There are two or three questions for each activity/section. The score ranges from 0 to 3, with higher scores translating into more severe disability [2].

WHOQOL-BREF

This index includes 26 items distributed into four domains related to the QoL: physical health, psychological health, social relationships, and environment. It also has one facet of overall QoL and general health (two additional items). Each domain score can be transformed to a 0-100 scale, with higher scores translating into a better QoL. The overall QoL and general health facets are scored on a five-point Likert scale and are presented from one to five, with higher scores translating into better outcomes [3].

APGAR

This index assesses how the family functioning is perceived at a given time. It is a self-administered questionnaire that includes five items, and each one evaluates a primary function of the family: adaptation, partnership, personal resource gradient/growth, affection, and resources/resolve. The score ranges from 0 to 10, where higher rates translate into a better family function [4].

DASS21

The scale is a set of three self-reported subscales that include seven items each, designed as a screening tool to assess the core symptoms of depression, anxiety, and stress. Depression, anxiety, and stress are calculated by summing the scores for the relevant items of each subscale. The DASS-21 is based on a dimensional rather than a categorical conception of psychological disorder. However, recommended cut-off scores for conventional severity labels (normal, mild, moderate, severe, and highly severe) have been published [5].

References

1. Pincus T, Bergman MJ, Yazici Y. RAPID3-an index of physical function, pain, and global status as “vital signs” to improve care for people with chronic rheumatic diseases. Bull NYU Hosp Jt Dis 2009; 67:2112–5. PubMed PMID: 19583557.

2. Bruce B, Fries JF. The Stanford Health Assessment Questionnaire: dimensions and practical applications. Health Qual Life Outcomes. 2003 Jun 9;1:20. PubMed PMID: 12831398; PubMed Central PMCID: PMC165587.

3. Development of the World Health Organization WHOQOL-BREF quality of life assessment. The WHOQOL Group. Psychol Med. 1998 May; 28 (3): 551–558. PubMed PMID: 9626712.

4. Gómez CFJ, Ponce RER. [A new proposal for the interpretation of Family APGAR]. (Spanish version). Aten Fam [Intenet].2010;17 (4):102-106. [cited 2024 Mar 24]. Available from: https://www.revistas.unam.mx/index.php/atencion_familiar/article/view/21348

5. Daza P, Novy DM, Stanley MA, Averill P. The Depression Anxiety Stress Scale-21: Spanish Translation and Validation with a Hispanic Sample. J Psychopathol Behav Assess. 2002;24: 195–205.
