# Supplementary material for: Perceived mistreatment in patients with rheumatic diseases: The impact of the underlying diagnosis
Source: PLoS One. 2024 Dec 30;19(12):e0316312. doi: 10.1371/journal.pone.0316312 (PMC11684605; doi:10.1371/journal.pone.0316312)
Supplement: S1 Table — (PDF) [file pone.0316312.s003.pdf]

Supplementary Table 1. Characteristics of the patients who were included in the RMD-MS validation process (S-1 and S-2).

	S-1 n=30	S-2 n=260
**Sociodemographic characteristics**		
Female sex*	27 (90)	237 (91.2)
Years of age	51.5 (38.9-59.3)	45 (33-54)
Years of formal education	11 (9-16)	12 (9-16)
Formal and non-formal job*	7 (23.3)	114 (43.8)
Married or Living with a partner*	13 (43.3)	120 (46.2)
Religious beliefs*	30 (100)	219 (84.2)
Medium-low socioeconomic status*	29 (96.7)	236 (91.1)
Family APGAR score (0-10 scale)	10 (6-10)	10 (8-12)
Patients with normal family function*	22 (73.3)	224 (86.2)
**Disease related-variables**		
Years of disease duration	12.5 (7.5-18.5)	10 (5-16)
RAPID-3 score (0-30 scale)	8.7 (3.3-14)	5.7 (1-11.5)
Patients with remission (RAPID-3 ≤3)	7 (23.3)	99 (38.1)
HAD-DI score (0-3 scale)	0.3 (0-1.3)	0.1 (0-1)
Patients with disability (HAQ-DI score >0.5)*	13 (43.3)	103 (39.6)
WHOQOL-BREF physical health score	51.8 (38.4-58)	53.6 (42.9-60.7)

WHOQOL-BREF mental health score	62.5 (54.2-70.8)	62.5 (50-70.8)
WHOQOL-BREF social relationship score	58.3 (50-66.7)	58.3 (50-75)
WHOQOL-BREF environment score	59.4 (46.9-68.8)	53 (46.8-65.6)
Rheumatic Diseases Comorbidity Index score	0 (0-1)	0 (0-1)
Patients with ≥ 1 comorbid condition*	15 (50)	131 (50.4)
One year-previous hospitalizations*	5 (16.7)	62 (23.8)
**Treatment-related variables**		
Immunosuppressive treatment*	21 (70)	216 (83.1)
N° of immunosuppressive drugs /patient[1]	1 (1-2)	1 (1-2)
Corticosteroids use*	8 (26.7)	90 (34.6)
**Mental health comorbidity**		
DASS21 score of ≥moderate severity[*,2]		
Depression	6 (20)	36 (13.8)
Anxiety	7 (23.3)	49 (18.8)
Stress	6 (20)	38 (14.6)

*Data presented as median (Q25-Q75) as otherwise indicated. *Number (%) of patients. [1]Among those who met the characteristic.[2]Lovibond SH, Lovibond PF. Manual for the Depression Anxiety & Stress Scales. 2nd ed. Sydney: Psychology Foundation; 1995.*
