# Supplementary material for: Perceived mistreatment in patients with rheumatic diseases: The impact of the underlying diagnosis
Source: PLoS One. 2024 Dec 30;19(12):e0316312. doi: 10.1371/journal.pone.0316312 (PMC11684605; doi:10.1371/journal.pone.0316312)
Supplement: S2 Table — (PDF) [file pone.0316312.s004.pdf]

Supplementary Table 2. Comparison of RMS-MS general score and dimension scores and the prevalence of mistreatment between patients with a normal family function and their counterparts.

	Patients with normal family function (n=252)	Patients with abnormal family function (n=8)	p-value
RMD-MS score	0.66±1.05	2.1±1.55	0.002
RMD-MS physical domain score	0.23±1.10	0.31±0.88	0.425
RMD-MS psychological domain score	1.74±2.91	6±3.02	0.000
RMD-MS neglect domain score	0.46±1.03	1.41±2.05	0.206
RMD-MS economic domain score	0.08±0.39	0.25±0.71	0.239
RMD-MS sexual domain score	0.26±0.95	0.83±1.54	0.077
N° (%) of patients with mistreatment	124 (49.2)	7 (87.5)	0.033

Data presented as mean ± SD *unless otherwise indicated. P-values are from the Mann-Whitney U test and* X^2 *test.*
