# Supplementary material for: Perceived mistreatment in patients with rheumatic diseases: The impact of the underlying diagnosis
Source: PLoS One. 2024 Dec 30;19(12):e0316312. doi: 10.1371/journal.pone.0316312 (PMC11684605; doi:10.1371/journal.pone.0316312)
Supplement: S3 Table — (PDF) [file pone.0316312.s005.pdf]

Supplementary Table 3. Spearman rank correlation coefficients (rho) between the RMD-MS score, specific dimensions scores of the DASS21, the family APGAR score, the HAQ-DI score, and the WHOQOL-BREF.

	Depression DASS21	Anxiety DASS21	Stress DASS21	APGAR	HAQ-DI	RAPID-3	Physical*	Psychological*	Social Relationship*	Environment*
**Rho**	0.251	0.240	0.217	0-0.272	0.122	0.141	-0.091	-0.242	-0.146	-0.169
**p-value**	≤0.0001	≤0.0001	≤0.0001	≤0.0001	0.049	0.023	0.144	≤0.0001	0.018	0.006

**Dimension from the WHOQOL-BREF.*
