# Supplementary material for: Perceived mistreatment in patients with rheumatic diseases: The impact of the underlying diagnosis
Source: PLoS One. 2024 Dec 30;19(12):e0316312. doi: 10.1371/journal.pone.0316312 (PMC11684605; doi:10.1371/journal.pone.0316312)
Supplement: S4 Table — (PDF) [file pone.0316312.s006.pdf]

Supplementary Table 4. Factorial Matrix.

	1	2	3	4	5
Have you been hit, for instance, by punches or kicks?	0.907				
Have you been shoved, shacked, or had your hair pulled?	0.832				
Have you had an object thrown at you intended to hurt you?	0.912				
Have you been assaulted with a knife, blade, gunfire, or another object?	0.917				
Have you felt humiliated or made fun of?			0.737		
Have you felt ignored or treated with indifference?			0.777		
Have you felt you have been isolated?			0.631		
Has anyone made you feel afraid?			0.686		
Has anyone made you feel less valued as a person?			0.754		
In general, have your decisions not been respected?	0.473				
Even having the necessary conditions, has anyone refused to provide you with essential things (clothes, food…) when needed?			0.549		
Even having the necessary conditions, has anyone refused to provide you with medications or required therapies when needed?			0.618		
Even having the necessary conditions, has anyone denied you help to go to a medical consultation or therapy when needed?			0.421		
Have you been denied protection even having the necessary conditions when you have felt that someone or something could harm you?				0.777	
Have you been forbidden to go out or to be visited?				0.797	
Have you been denied access to your home?				0.789	
Have you been kicked out of the house?				0.523	

Has anyone managed or is anyone managing your money without your consent or pressing you to assign it to some family expenses?		0.812			
Has your money been taken from you?		0.804			
Has anyone taken any of your belongings without your permission?		0.728			
Have any of your properties been sold without your consent?		0.920			
Have you been pressured so that you no longer own your house or any other property?		0.917			
Have you been forced to have sex even if you did not want to?					0.886
Has anyone touched your body, including your genitals, without your consent?					0.849
Have you felt sexual rejection from your partner?					0.764
