# Supplementary material for: Perceived mistreatment in patients with rheumatic diseases: The impact of the underlying diagnosis
Source: PLoS One. 2024 Dec 30;19(12):e0316312. doi: 10.1371/journal.pone.0316312 (PMC11684605; doi:10.1371/journal.pone.0316312)
Supplement: S5 Table — (PDF) [file pone.0316312.s007.pdf]

Supplementary Table 5. Impact of removing items on RMD-MS Cronbach's alpha.

RMD-MS Dimensions and items	Cronbach's alpha value if the item is removed					
	Global scale	D-1	D-2	D-3	D-4	D-5
**D.1-Physical**						
Have you been hit, for instance, by punches or kicks?	0.878	0.927				
Have you been shoved, shacked, or had your hair pulled?	0.878	0.959				
Have you had an object thrown at you intended to hurt you?	0.878	0.919				
Have you been assaulted with a knife, blade, gunfire, or another object?	0.878	0.927				
**D.2-Psychological**						
Have you felt humiliated or made fun of?	0.880		0.807			
Have you felt ignored or treated with indifference?	0.878		0.785			
Have you felt you have been isolated?	0.881		0.835			
Has anyone made you feel afraid?	0.879		0.835			
Has anyone made you feel less valued as a person?	0.875		0.804			
**D.3-Neglect**						
In general, have your decisions been respected?	0.881			0.790		
Even having the necessary conditions, has anyone refused to provide you with essential things (clothes, food…) when needed?	0.879			0.765		
Even having the necessary conditions, has anyone refused to provide you with medications or required therapies when needed?	0.880			0.782		
Even having the necessary conditions, has anyone denied you help to go to a medical consultation or therapy when needed?	0.885			0.783		

Have you been denied protection even having the necessary conditions when you have felt that someone or something could harm you?	0.880			0.749		
Have you been forbidden to go out or to be visited?	0.879			0.759		
Have you been denied access to your home?	0.880			0.756		
Have you been kicked out of the house?	0.880			0.770		
**D.4-Economic**						
Has anyone managed or is anyone managing your money without your consent or pressing you to assign it to some family expenses?	0.879				0.886	
Has your money been taken from you?	0.879				0.903	
Has anyone taken any of your belongings without your permission?	0.881				0.888	
Have any of your properties been sold without your consent?	0.884				0.869	
Have you been pressured so that you no longer own your house or any other property?	0.885				0.866	
**D.5-Sexual**						
Have you been forced to have sex even if you did not want to?	0.887					0.634
Has anyone touched your body, including your genitals, without your consent?	0.887					0.667
Have you felt sexual rejection from your partner?	0.888					0.862

D=Dimension
